# Predicting Cell Differentiation in Mechanically Stimulated Biphasic Osteochondral Scaffolds Using Fluid–Structure Interaction Modelling

**DOI:** 10.3390/bioengineering13070809

**Published:** 2026-07-15

**Authors:** Pedram Azizi, Ursula van Rienen, Hermann Seitz

**Affiliations:** 1 Chair of Microfluidics, Faculty of Mechanical Engineering and Marine Technology, University of Rostock, 18059 Rostock, Germany; hermann.seitz@uni-rostock.de; 2 Chair of Electromagnetic Field Theory, Faculty of Computer Science and Electrical Engineering, Institute of General Electrical Engineering, University of Rostock, 18059 Rostock, Germany; ursula.van-rienen@uni-rostock.de; 3 Department of Life, Light and Matter, University of Rostock, 18059 Rostock, Germany; 4 Department of Ageing of Individuals and Society, Interdisciplinary Faculty, University of Rostock, 18059 Rostock, Germany

**Keywords:** osteochondral defects, biphasic scaffolds, tissue engineering, mechanical stimulation, fluid–structure interaction, computational fluid dynamics, finite element analysis, in silico, cell differentiation

## Abstract

Osteochondral defects, involving both articular cartilage and subchondral bone, can lead to joint degeneration and osteoarthritis. Recent advances in 3D-printed biphasic scaffolds offer promising opportunities to recreate physiological microenvironments for tissue regeneration. In tissue engineering, these scaffolds can be mechanically stimulated to promote targeted cartilage and bone formation. While computational models have been widely used to study mechanically induced cellular responses in monophasic scaffolds, time-dependent modelling of biphasic osteochondral systems remains relatively scarce. In this study, a fluid–structure interaction (FSI) framework coupled with a mechanoregulatory algorithm was developed to predict mechanically induced early-stage mesenchymal stem cell (MSC) differentiation in biphasic open-porous osteochondral scaffolds comprising chondral and bone layers designed for direct ink writing (DIW). In a second model, an interfacial barrier layer representing the native osteochondral interface was integrated. Dynamic compressive loading (1 Hz, 2.5% strain) was applied. The simulations predicted region-specific differentiation patterns in both the chondral and subchondral bone regions. In the scaffold without a barrier layer, approximately 68.9% of MSCs in the chondral layer and 93.4% of MSCs in the bone layer underwent chondrogenic and osteogenic differentiation, respectively. Incorporation of the barrier layer caused only minor changes, reducing predicted cartilage and bone differentiation by approximately 1.5% and 3.9%, respectively. Overall, this study highlights the capability of computational modelling to predict mechanobiological responses in complex osteochondral systems and support scaffold design and effective mechanical stimulation protocols.

## 1. Introduction

Injuries and diseases affecting articular cartilage and the osteochondral junction are clinically significant because cartilage has limited self-healing capacity, leading to joint instability, pain, progressive degeneration, and decreased quality of life worldwide [1]. Over recent decades, osteochondral tissue engineering has advanced rapidly, demonstrating great promise as a viable treatment for osteochondral disorders [2].

The osteochondral unit includes articular cartilage and subchondral bone, each with unique biomechanical properties that work together to ensure proper load distribution and maintain joint health [3]. The chondral layer, being the most superficial layer of the osteochondral unit, is subjected to a greater number of force vectors, while the subchondral bone provides compressive strength to the osteochondral unit and features a low elastic modulus [4]. The native osteochondral unit exhibits a continuous transition from cartilage to bone, involving changes in composition, mineralisation, porosity, and stiffness, which inspires scaffold designs ranging from monophasic to gradient and triphasic structures [1]. The progress of 3D-printed biphasic scaffolds for repairing osteochondral defects has improved considerably in recent years [5]. Extrusion-based bilayer scaffold designs have enabled the development of separate cartilage and bone regions with customised stiffness, thereby creating site-specific mechanical conditions for encapsulated cells [6,7]. Direct ink writing (DIW) is an extrusion-based additive manufacturing technique that enables the fabrication of complex 3D scaffolds with controlled architecture, porosity, and composition, offering high reproducibility and versatility for tissue engineering applications [8]. For example, DIW can be used to fabricate conductive hydrogel-based biomedical scaffolds [9] or 3D-printed hydroxyapatite bone scaffolds, with polycaprolactone (PCL) serving as a binder [10].

Recent experimental studies on 3D-printed osteochondral scaffolds have demonstrated substantial progress in promoting distinct cartilage and bone tissue formation in both in vitro and in vivo models. For example, an in vitro study by Khoshnood et al. [11] developed a novel 3D-printed biphasic osteochondral scaffold comprising a PCL/laponite composite for the bone layer and methylsulfonylmethane-loaded PCL/chitosan for the cartilage layer, which promoted region-specific differentiation of bone marrow-derived MSCs under static culture. The bone layer enhanced osteogenic differentiation, including a 3.3-fold increase in COL I expression, while the cartilage layer promoted chondrogenic differentiation, as reflected by a 2.51-fold increase in COL II expression. Among recent in vivo studies, Qin et al. [12] implanted a customised 3D-printed biphasic scaffold into full-thickness osteochondral defects in rabbits and demonstrated simultaneous regeneration of hyaline cartilage and subchondral bone. The scaffold significantly improved bone formation, as indicated by increased bone volume and trabecular number, together with reduced trabecular separation, while histological and immunohistochemical analyses confirmed enhanced cartilage maturation through increased proteoglycan and collagen II deposition and reduced collagen I expression.

Computational methods in tissue engineering substantially reduce experimental time and not only improve scaffold design processes but also broaden design capabilities, offering researchers a versatile and efficient framework for optimising scaffold architecture [13]. Among in silico methods, finite element (FE) analysis has been widely applied to model biomedical scaffolds and to study how design parameters influence their mechanical performance. For example, FE simulations of extrusion-based 3D-bioprinted PCL scaffolds showed that both scaffold infill geometry and alginate-hydrogel incorporation substantially influence mechanical behaviour, underscoring the importance of scaffold architecture and material composition in tailoring scaffold mechanics [14]. Most studies have focused on monophasic scaffolds. However, one of the few FE studies on biphasic osteochondral scaffolds was conducted by Choe et al. [15], who investigated fibril-based interfacial patterns to optimise the biomechanical performance of osteochondral scaffolds under compressive and shear loading. This study used a 3D, stationary solid-mechanics model to demonstrate that a mechanically interlocking interface enhances load-bearing and regenerative performance, but it did not predict cell differentiation or other mechanobiological responses. Additionally, computational fluid dynamics (CFD) has demonstrated strong capability to quantify the fluidic characteristics of scaffolds. As an example of such an application, a CFD study of diamond and gyroid scaffolds with porosities ranging from 50% to 80% showed that gyroid structures exhibited lower pressure drops, higher permeability, and favourable wall shear stress (WSS) distributions, making them more suitable for orthopaedic implants and tissue regeneration applications [16]. Although FE and CFD simulations are powerful tools for analysing the structural and fluid-dynamic behaviour of biological scaffolds, their independent application cannot fully capture the mechanobiological microenvironment, in which scaffold deformation and fluid-induced shear jointly influence cell behaviour. A more comprehensive numerical approach is fluid–structure interaction (FSI) modelling, which captures the coupled effects of fluid-induced stresses and solid mechanical responses within scaffolds and their consequent influence on seeded cells. Computer-aided design (CAD) enables precise control over the architecture of 3D-printed scaffolds. FSI simulations have been utilised to investigate how CAD-controlled structural design parameters, such as pore size and shape, affect cellular mechanical stimulation, including WSS and mechanical strain [17]. Ferroni et al. [18] demonstrated the necessity of FSI modelling for accurately representing fluid-induced stimuli in complex porous scaffolds. They showed that FSI predicted shear stress values well below damaging thresholds for cardiac cells, whereas simplified laminar flow models overestimated these values by up to 1000-fold. Zhao et al. [19] developed a multiscale model linking scaffold-scale CFD with a cell-scale FSI model and reported an increase in average cell WSS from 1.53 to 5.74 mPa during tissue growth, while mechanical strain remained negligible. Although the model enabled location- and morphology-specific quantification of mechanical stimuli, it considered only a limited number of scaffold locations, and both the scaffold and the cell/extracellular matrix regions were modelled as single-phase materials. Another recent multiscale computational study integrated a global FSI model with a local cell-scale FE model to investigate the mechanical responses of osteoblasts in trabecular bone and gyroid scaffolds [20]. The authors reported WSS between 10mPa and 80mPa across most of the scaffold and the 3D-modelled bone surface, while site-specific cell-level values remained below 60mPa. Although their approach provided valuable insights into cell-level mechanical responses, it was developed for a single-phase bone scaffold under steady loading conditions, with analyses performed at only a limited number of scaffold locations. Samanta et al. [21] developed a multiscale computational framework to evaluate the mechanical stimulation of cells at selected locations within a monophasic porous titanium alloy scaffold subjected to fluid flow. A global one-way FSI model was first used to predict scaffold deformation under flow conditions, followed by a local FE sub-model to quantify cell deformation based on the global response. The maximum cell strain occurred at the cell–substrate contact region, reaching approximately $4\times 10^{-5}$ for an inlet velocity of $1\;\mathrm{mm}\;s^{-1}$. In contrast to previous FSI studies, which primarily focused on single-phase scaffolds, the present study investigates a biphasic osteochondral scaffold under transient loading conditions and predicts cell differentiation throughout the scaffold.

Most advanced studies have focused on testing and modelling articular cartilage and subchondral bone separately, but there is a lack of research on the osteochondral unit as a unified tissue [22]. Chondrocytes and osteoblasts need distinct microenvironments, and biphasic osteochondral scaffolds more effectively mimic physiological conditions than monophasic scaffolds by supporting the growth and differentiation of both cell types [2]. Therefore, developing a coherent, experimentally validated, integrated model within a unified framework to realistically simulate osteochondral biomechanics has been a major challenge [23].

In this study, a model of a biphasic osteochondral scaffold comprising porous chondral and subchondral bone layers was developed and numerically investigated. The model was designed to analyse mechanical stimulation via dynamic compression of an osteochondral scaffold seeded with mesenchymal stem cells (MSCs) in the context of tissue engineering for the treatment of osteochondral defects. The model accounts for both fluidic and mechanical effects through an FSI approach, thereby capturing the combined biomechanical environment within the scaffold. Based on the mechanical stimuli acting on cells at the scaffold surface, the model predicted the direction of cell differentiation in both the chondral and bone layers. The integration of an interfacial barrier layer into 3D-printed biomimetic scaffolds was also considered, as such layers are frequently implemented in osteochondral scaffolds to maintain distinct biological environments between cartilage and bone. This function helps prevent vascular invasion into cartilage and undesired calcification [24]. Hence, we studied the influence of this interfacial layer by designing two scaffold configurations using CAD: one with an interfacial barrier layer and one without. Both configurations were designed to be manufacturable by direct ink writing (DIW) 3D printing.

## 2. Materials and Methods

### 2.1. Geometry

**Figure 1:**
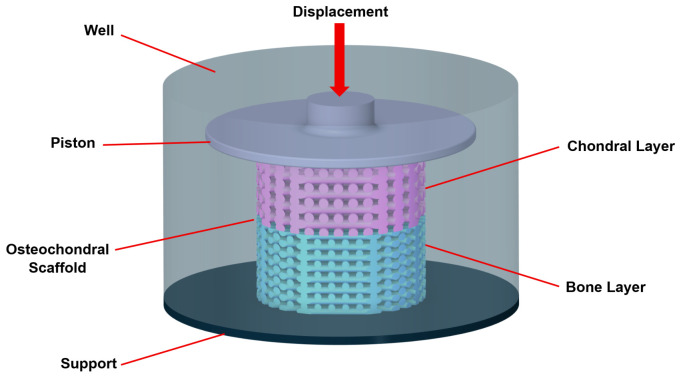
The geometry of the numerical model.

The model geometry comprises an osteochondral scaffold placed within a well of a conventional 12-well plate. A piston is positioned above the scaffold and can move vertically within the well to compress it, as shown in Figure 1. The well’s height and diameter are 13.3 mm and 21 mm, respectively.

**Figure 2:**
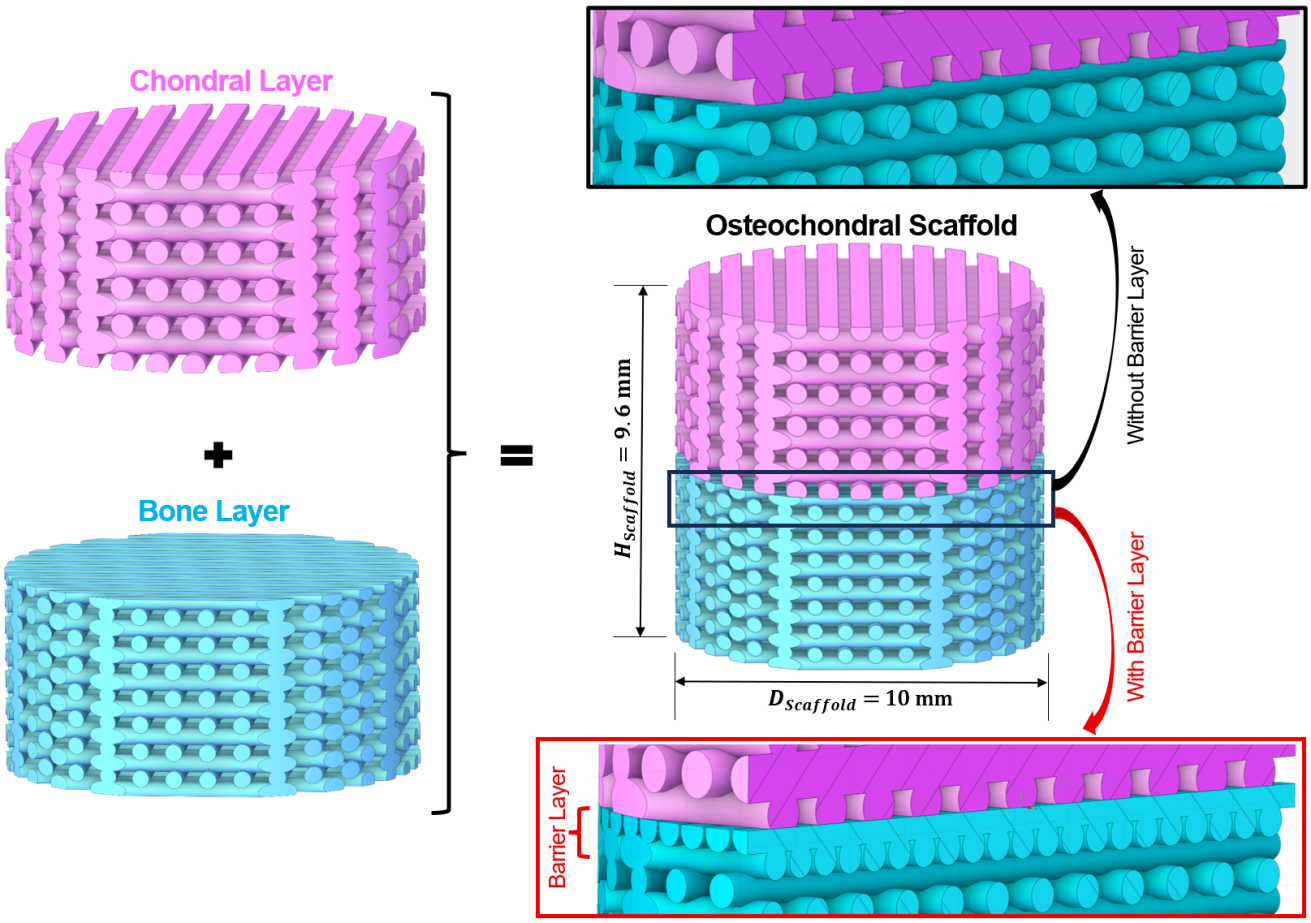
Combination of the chondral and bone layers to form biphasic osteochondral scaffolds. The separate chondral and bone layers are shown on the left, while their combined configuration forming the osteochondral scaffold is presented on the right. The upper-right part shows a zoomed-in view of the scaffold without a barrier layer at the chondral–bone interface, whereas the lower-right part shows the scaffold with a barrier layer. In the latter case, the barrier layer is formed within the first two sublayers of the bone layer.

**Figure A1:**
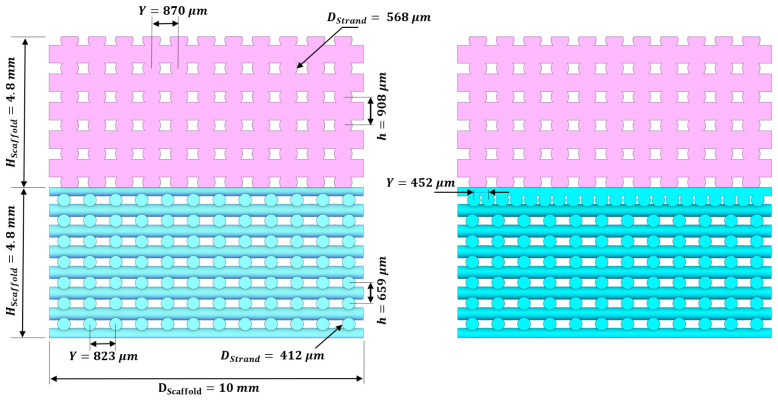
The dimensions of the osteochondral scaffold are shown on the left without the barrier layer and on the right with it. The only difference in the scaffold with the barrier layer is the horizontal span (*Y*) in the two uppermost sublayers of the bone layer.

The solid domain includes the piston, osteochondral scaffold, and support, whereas the fluid domain is obtained by subtracting the solid domain from the well volume. The biphasic osteochondral scaffolds consist of two main regions: the chondral layer and the bone layer. Although the scaffolds investigated in this numerical study have not yet been manufactured for in vitro experiments, both layers were designed to be printable by DIW using different material inks, namely hydrogel-based inks for the chondral layer and biopolymer-based inks for the bone layer. In the lower part of the osteochondral scaffold, the bone layer is positioned, with the chondral layer placed on top of it, as shown in Figure 2. The chondral and bone layers were aligned such that the strands at the bottom of the chondral layer were oriented at 90° relative to the strands on the top of the bone layer. The layers were independently designed in SOLIDWORKS 2021 (Dassault Systèmes SolidWorks Corporation, Waltham, MA, USA) and then imported into ANSYS Discovery 2024 R2 (ANSYS Inc., Canonsburg, PA, USA) to combine them and form the solid and fluid domains. A shared topology was employed among the chondral layer, bone layer, and fluid domain to generate a conformal mesh for these components in ANSYS Discovery. The structural design of the chondral layer was selected from our previous numerical research study [25]. In that study, the monophasic scaffold S2-H11-V11, hereafter referred to as the monophasic cartilage reference scaffold, was identified as a suitable design for enhancing chondrogenic cell differentiation in the chondral layer, based on a detailed analysis of its porosity and pore dimensions. This layer, designed to be fabricated from alginate–gelatin (ADA–GEL) using DIW, consisted of 11 printed sublayers, each comprising 11 strands, with a porosity of 39% (Figure A1 in Appendix A). The strand diameter ($D_{Strand}$) of the chondral layer was selected as 568 μm, which has been experimentally demonstrated as a printable strand diameter for cross-linked hydrogel scaffolds [26]. Furthermore, its pore size of 302 μm falls within the optimal range of 200 μm to 400 μm reported for 3D-printed ADA-GEL scaffolds, providing a favourable balance between mechanical performance and pore architecture for tissue engineering applications [27]. For the bone layer, the dimensions were selected based on pore sizes and strand diameters suitable for fabrication using the DIW method (Figure A1 in Appendix A). This layer consisted of 15 printed sublayers, each comprising 12 strands, with a porosity of 52%. $D_{Strand}$ of the bone layer was set to 412 μm, which closely matches the experimentally achieved strand width range of 420–521 μm reported in a recent study on biodegradable subchondral bone scaffolds fabricated from PCL using DIW [28]. In the same study, pore sizes ranging from 100 μm to 1000 μm were successfully fabricated, encompassing the bone-layer pore size of 411 μm adopted in the present study. Recent studies have shown that PCL-based composite scaffolds with nozzle diameters and pore sizes similar to those selected in the present study can be fabricated by extrusion-based 3D printing for bone tissue engineering applications [29]. The final osteochondral scaffold has a diameter ($D_{Scaffold}$) of 10 mm and a height ($H_{Scaffold}$) of 9.6 mm, as demonstrated in Figure 2. $D_{Scaffold}$ is clinically relevant, as it corresponds to a commonly used size in large-animal in vivo models for osteochondral defect repair [30]. This scaffold dimension has also been associated with enhanced bone ingrowth and superior hyaline-like cartilage regeneration in multilayered additively manufactured osteochondral scaffolds [31]. To understand how a barrier layer affects cell differentiation, a modified version of the osteochondral scaffold was also designed. The only difference in this design is the reduced distance between the strands in the first two sublayers (the barrier layer) of the bone layer. The horizontal span in the barrier sublayers is 452 μm, reducing the distance between adjacent strands to 40 μm (Figure A1 in Appendix A). For the sublayers beneath the barrier layer, the strand spacing remains the same as in the model without a barrier layer, with a distance of 411 μm between adjacent strands.

### 2.2. Mesh

**Figure 3:**
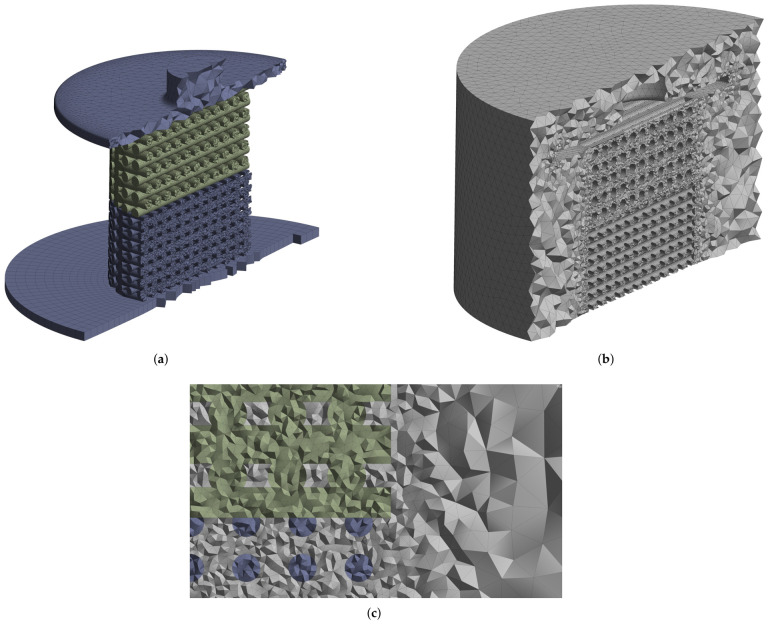
Mesh details for both solid and fluid domains: (**a**) Meshing of the FE model. (**b**) Meshing of the CFD model. (**c**) Conformal mesh structure.

The mesh was generated using ANSYS Meshing 2024 R2, with conformal meshing applied to both solid (Figure 3a) and fluid (Figure 3b) domains. Following a mesh independence study of three mesh types, namely coarse, medium, and fine, as described in Appendix A.2 of Appendix A, the medium mesh was selected. To better capture velocity gradients near the curved surfaces of the CFD domain and enhance solution accuracy, the mesh was finer around the strands, as shown in Figure 3b. The conformal meshing approach allowed for matching the FE and CFD nodes at each element on the scaffold surface, enabling the calculation of structural and fluidic properties at the same node (Figure 3c). Additional mesh details are provided in Appendix A.2 of Appendix A.

The simulation setup for the monophasic cartilage reference scaffold is detailed in our earlier work [32]. Below, we briefly describe the biphasic scaffold setup, which differs slightly from the one used in the mentioned study.

### 2.3. FE Model and Material Properties

**Figure 4:**
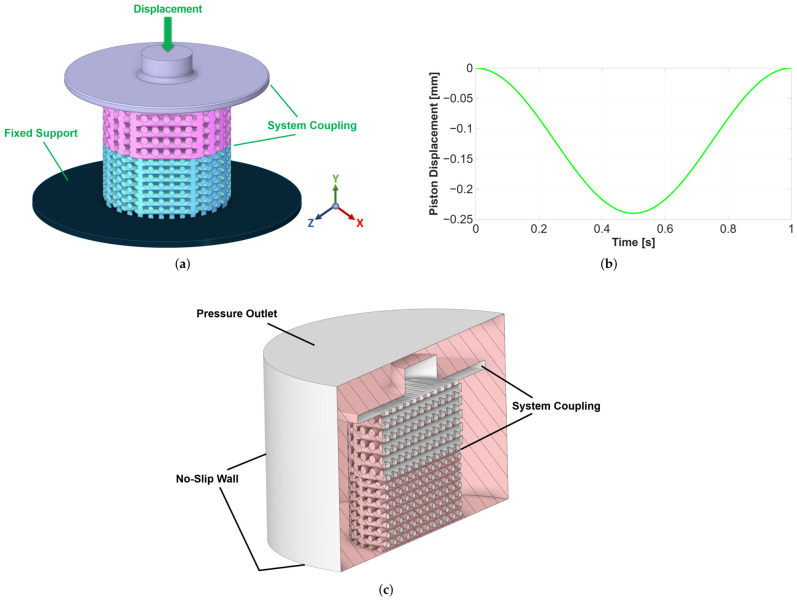
Boundary conditions of the FSI model: (**a**) Boundary condition of the FE model. (**b**) Displacement curve of the piston during one load cycle at a frequency of 1 Hz. (**c**) Boundary condition of the CFD model.

The FE model was developed using ANSYS Transient Structural 2024 R2. A frictional contact with a coefficient of 0.2 was set between the piston and the top surface of the osteochondral scaffold. The mechanical compression was simulated based on the piston’s displacement, which was restricted to vertical movement along the Y direction, as shown in Figure 4a. The displacement followed a sinusoidal pattern with an amplitude of 2.5% relative to the scaffold height and a frequency of 1 Hz, thereby mimicking the pace of human gait [33], as depicted in Figure 4b. As the osteochondral scaffold comprises a deformable soft component, represented by the chondral layer, and an almost rigid component, represented by the bone layer, the applied displacement is mainly accommodated by compression of the chondral layer, while deformation of the bone layer remains negligible. This results in an effective compression amplitude of 5% in the chondral layer, reflecting the boundary condition used by [25], which guided the design of an optimal hydrogel scaffold for cartilage cell differentiation. The piston and scaffold surfaces were designated as the system coupling region for data exchange between the FE and CFD models (Figure 4a). The support was fixed, with its contact to the bottom of the osteochondral scaffold modelled as frictional with a coefficient of 0.2. Since shared topology was used between the chondral and bone layers, a separate contact definition was not necessary for their joint. Consequently, these layers were prevented from relative motion, with nodes merged to form a continuous mesh at their interface.

The material properties of the chondral layer were modelled to match the mechanical behaviour of pure ADA-GEL, including an average Young’s modulus of 11.3 kPa, a compression–tension asymmetry ratio of 2, a nonlinearity parameter of approximately −1.3, a non-collagenous fibrous microstructure, and stress relaxation with hysteresis [34]. The experimental data from this study were integrated into a one-term Ogden model, appropriate for hyperelastic materials. The Ogden model’s general form is described as [35]:

(1) $$W=\sum_{i=1}^{N}\frac{\mu_{i}}{\alpha_{i}}\left({\bar{\lambda }}_{1}^{\alpha_{i}}+{\bar{\lambda }}_{2}^{\alpha_{i}}+{\bar{\lambda }}_{3}^{\alpha_{i}}-3\right)+\sum_{i=1}^{N}\frac{1}{d_{i}}{\left(J-1\right)}^{2i}$$

where *W* is the strain-energy potential, $\lambda_{1}$, $\lambda_{2}$, and $\lambda_{3}$ are principal stretch ratios, *J* represents the volume ratio indicating the ratio of deformed to undeformed volume of the material, and $\mu_{i}$, $\alpha_{i}$, and $d_{i}$ are user-defined material constants. These constants were obtained from compression–tension measurements [34], with $\mu_{1}=-5.8$ kPa and $\alpha_{1}=-1.3$. Furthermore, it was assumed that the hydrogel is incompressible, with $d_{1}=0$.

The material properties of the bone layer were defined based on PCL, which exhibits mechanical properties similar to those of cancellous porous bone. Moreover, it is suitable for 3D printing using DIW and can be combined with bioactive materials [28]. The PCL was characterised by a Young’s modulus of 299.3 MPa and a Poisson’s ratio of 0.3 [36]. Since the chondral layer was modelled as non-viscoelastic and the bone layer was assigned time-independent material properties, the simulations were limited to a single loading cycle. Under these assumptions, additional loading cycles would not alter the material response and would therefore not affect the resulting mechanical stimuli.

### 2.4. CFD Model

The CFD simulations of fluid flow within and around the scaffold were conducted using ANSYS Fluent 2024 R2. A pressure outlet boundary condition was applied at the top of the well, while the other surfaces were modelled as stationary walls with no-slip conditions, as shown in Figure 4c. The interface between the fluid and structural models, namely the piston and scaffold, employed a dynamic mesh approach to receive the mechanical deformation from the FE model as the CFD boundary condition. Dynamic mesh zones were defined as system coupling, utilising both smoothing via the diffusion method and remeshing. The culture medium inside the fluid domain was considered incompressible and Newtonian, with a density of $1000\;\mathrm{kg}\;m^{-3}$ and a dynamic viscosity of $1.45\times 10^{-3}\;\mathrm{Pa}\cdot s$. Given a maximum Reynolds number of approximately 1, calculated based on the strand diameter, the flow was assumed to be laminar. The solver used was pressure-based, with pressure–velocity coupling via a coupled scheme that employed a second-order implicit transient formulation. Convergence was ensured by setting the residuals for continuity and velocity to $10^{-4}$.

### 2.5. FSI Model

FE and CFD models were coupled using the System Coupling Module in ANSYS Workbench 2024 R2. The FSI simulation used a one-way, transient co-simulation approach, transferring data from the FE model to the CFD model. The data exchange occurred only from the structural to the fluid model at the interface because the fluid flow was induced by a prescribed mechanical displacement and was therefore assumed to have a negligible feedback effect on the mechanical movement. A sensitivity study on time-step size was conducted using values of 0.005 s and 0.01 s to evaluate how the results depend on this parameter. Both outcomes were very similar. Therefore, a time step of 0.01 s was chosen to speed up the simulations.

### 2.6. Mechanoregulation Algorithm

To evaluate cell differentiation on the surface of the osteochondral scaffold, the mechanoregulation theory by Prendergast et al. [37] was used. This theory proposes that a combination of mechanical strain and fluid flow can predict the differentiation outcomes of MSCs under mechanical stimulation. A modified version of this theory [38] was applied in the current study, as follows:

(2) $$S=\frac{\mathrm{OSS}}{a}+\frac{\mathrm{WSS}}{b}$$

where S represents stimuli, WSS is wall shear stress, OSS is octahedral shear strain, and a and b are the constants in the equation, with values of 0.0375 and 10mPa, respectively. The S value derived from this calculation predicted the cell phenotype, as reported in Table 1. The stimulus S acts on MSCs over time and regulates their differentiation in a magnitude-dependent manner [39]. High values of S promote differentiation into fibroblasts and the formation of fibrous connective tissue, intermediate values stimulate differentiation into chondrocytes and cartilage formation, and low values favour osteogenic differentiation and bone formation, with very low stimuli causing resorption [39]. OSS in Equation (2) was determined from the FE model using the following formula:

(3) $$\mathrm{OSS}=\frac{2}{3}\sqrt{{\left(\varepsilon_{1}-\varepsilon_{2}\right)}^{2}+{\left(\varepsilon_{2}-\varepsilon_{3}\right)}^{2}+{\left(\varepsilon_{3}-\varepsilon_{1}\right)}^{2}}$$

where $\varepsilon_{1}$, $\varepsilon_{2}$, and $\varepsilon_{3}$ are the elastic principal strains. And WSS was computed from the CFD model as follows:

(4) $$\mathrm{WSS}=\mu \frac{\partial u}{\partial n}$$

where μ is the dynamic viscosity, and $\frac{\partial u}{\partial n}$ represents the normal gradient of fluid velocity on the walls.

The conformal meshing approach enabled us to calculate the parameter S at each numerical node on the interface (scaffold) and predict cell differentiation at these points. A MATLAB R2025b (MathWorks Inc., Natick, MA, USA) script was developed to match the FE nodes and the computed OSS from the FE model with the CFD nodes and their respective WSS values. Subsequently, the parameter S was computed at each matched node using the OSS and WSS values obtained from the FE and CFD models, respectively (Equation (2)). Since the simulations were transient, it was essential to identify the specific time point when cell differentiation occurred. This was determined by identifying the time at which $S_{Avg}$ reached its maximum during the simulation. The $S_{Avg}$ at each time was calculated using Equation (2) with two parameters: firstly, the area-weighted average of wall shear stress across the entire scaffold surface ($WSS_{Avg}$), and secondly, the arithmetic mean of the octahedral shear strain at all FE nodes on the scaffold surface ($OSS_{Avg}$). By substituting $OSS_{Avg}$ and $WSS_{Avg}$ into Equation (2) as OSS and WSS, respectively, we obtained the $S_{Avg}$ at each time point and identified its maximum during a loading cycle.

## 3. Results

The simulation results for the osteochondral scaffolds are presented separately for the chondral and bone layers, enabling a more detailed, layer-specific analysis.

### 3.1. $WSS_{Avg}$ and $OSS_{Avg}$ on the Chondral and Bone Layers

**Figure 5:**
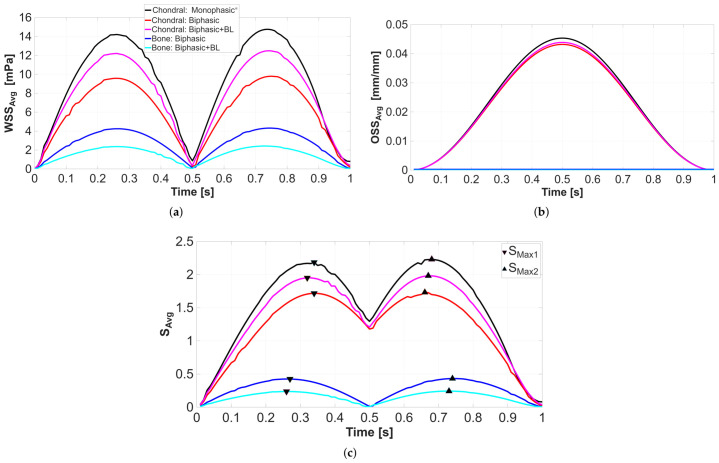
Averaged simulation results across the entire surface of the scaffolds during a loading cycle: (**a**) $WSS_{Avg}$ on the chondral and bone layers, compared with a previously optimised monophasic cartilage reference scaffold. In the legend, “Chondral: Monophasic” refers to the monophasic cartilage reference scaffold, while “Chondral: Biphasic + BL” and “Bone: Biphasic + BL” refer to the chondral and bone layers of the osteochondral scaffold with a barrier layer. “Chondral: Biphasic” and “Bone: Biphasic” denote the chondral and bone layers of the osteochondral scaffold, without a barrier layer. (**b**) The average OSS ($OSS_{Avg}$) during a loading cycle over the whole surface. (**c**) The average stimulus value ($S_{Avg}$) across the entire surface, with peaks marked as the piston moves downward ($S_{Max1}$) and upward ($S_{Max2}$). * Results for the monophasic cartilage reference scaffold are from the previous numerical study [25].

Figure 5 compares the average values of fluidic forces and mechanical deformation on both layers. In Figure 5a, $WSS_{Avg}$ on the entire surface of the chondral and bone layers for both scaffold cases with and without a barrier layer is compared to the monophasic cartilage reference scaffold used in our previous study [25]. As shown in Figure 5a, the highest $WSS_{Avg}$ values were observed in the monophasic cartilage reference scaffold, followed by the chondral layer of the osteochondral scaffold with a barrier layer, with a range from 0 to 12.49mPa. This was followed by the chondral layer without a barrier layer, which exhibited values ranging from 0 to 9.80mPa. In contrast, the bone layer with a barrier layer recorded the lowest values, ranging from 0 to 2.42mPa, while the case without a barrier layer ranged from 0 to 4.31mPa. During piston movement in a loading cycle (Figure 4b), the $WSS_{Avg}$ exhibited two peaks within a cycle: one during piston compression and another during piston release, as illustrated in Figure 5a. The $OSS_{Avg}$ on the scaffold surface during loading (Figure 5b) revealed that only the chondral layer of the osteochondral scaffolds, similarly to the monophasic cartilage reference scaffold, experienced noticeable deformation and shear strain on its surface. As expected, the maximum $OSS_{Avg}$ (approximately 0.045) occurred when the piston reached its lowest position (t=0.5s). The $S_{Avg}$ curves, calculated by substituting $WSS_{Avg}$ and $OSS_{Avg}$ into Equation (2), are plotted in Figure 5c. Their behaviour closely resembles that of the $WSS_{Avg}$ curves shown in Figure 5a, where the chondral layer and the monophasic cartilage reference scaffold experience higher average mechanical stimulation compared to the bone layer. The maximum $S_{Avg}$ values appeared during piston compression ($S_{Max1}$) and release phases ($S_{Max2}$), with $S_{Max2}$ slightly exceeding $S_{Max1}$. The time points at which $S_{Max2}$ occurred were considered the moments when cell differentiation on the scaffolds was predicted.

### 3.2. Mechanical Microenvironment of the Chondral Layer

**Figure 6:**
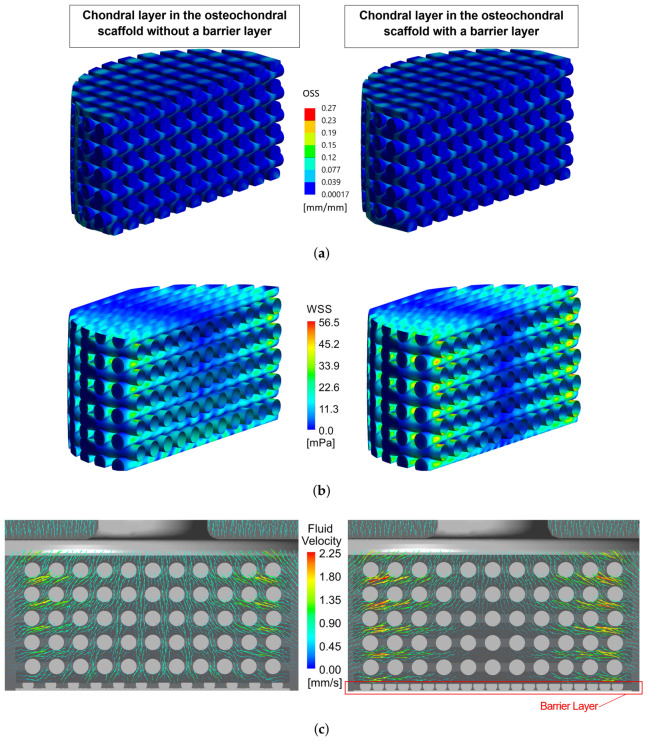
Post-processing of simulation results for the chondral layers of the osteochondral scaffolds, both without (**left**) and with a barrier layer (**right**): (**a**) Structural strain comparison (OSS). (**b**) Fluid-induced shear stress comparison (WSS). (**c**) Velocity vectors within the fluid domains, coloured according to the fluid velocity magnitude using a linear colour scale. The legend in each subfigure remains the same in both cases, with and without the barrier layer, to make comparisons clearer. All results are shown at a time when cell differentiation was predicted (without barrier layer: 0.66s; with barrier layer: 0.67s).

**Figure 7:**
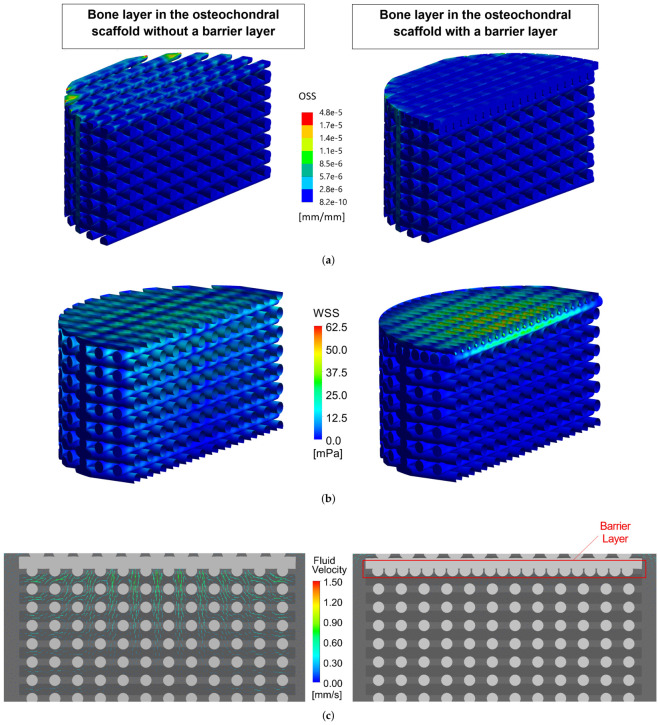
Post-processing of simulation results for the bone layer of the osteochondral scaffolds, both without (**left**) and with a barrier layer (**right**): (**a**) Structural strain comparison (OSS). (**b**) Fluid-induced shear stress comparison (WSS). (**c**) Velocity vectors within the fluid domains, coloured according to the fluid velocity magnitude using a linear colour scale. The legend in each subfigure remains the same in both cases, with and without the barrier layer, to make comparisons clearer. All results are shown at a time when cell differentiation was predicted (without barrier layer: 0.74s; with barrier layer: 0.73s).

Post-processing of simulation results to analyse the mechanical stimulation mechanism is demonstrated in Figure 6 and Figure 7. The chondral layers of the osteochondral scaffolds, both with and without a barrier layer, are compared in Figure 6. The OSS values on the surfaces of the chondral layers, with and without a barrier layer, are compared in Figure 6a. The local maximum and minimum OSS values on the scaffold surface were not identical. For the scaffold without a barrier layer, OSS ranged from $1.72\times 10^{-4}$ to 0.27, whereas the scaffold with a barrier layer exhibited corresponding values between $2.14\times 10^{-4}$ and 0.16 (Figure 6a). Nevertheless, the OSS distributions were very similar in both cases, resulting in only a marginal difference in $OSS_{Avg}$, which was 0.033 for the scaffold without a barrier layer and 0.032 for the scaffold with a barrier layer, as also illustrated in Figure 5b. Comparing the WSS on the scaffold surfaces reveals that the chondral layer with a barrier layer experienced higher WSS values, as illustrated in Figure 6b. On the surface of the chondral layer, WSS ranged between 0.08mPa and 43.48mPa for the scaffold without a barrier layer and between 0.04mPa and 56.41mPa for the scaffold with a barrier layer (Figure 6b). The $WSS_{Avg}$ on the surface of the chondral layer with a barrier layer was 11.21mPa, compared to 8.52mPa without a barrier layer. Furthermore, Figure 5 also demonstrates that the barrier layer elevates the $WSS_{Avg}$ on the chondral layer during a loading cycle. The fluid flow through the pores of the chondral layer is illustrated in Figure 6c. In the osteochondral scaffold with a barrier layer, less medium flows into the central regions of the chondral layer than in the scaffold without a barrier layer. All results shown in Figure 6 are recorded at the moment when $S_{Max2}$ occurred (see Figure 5c), during the upward movement of the piston. For the scaffold without a barrier layer, this time is 0.66s, while for the other scaffold, it is 0.67s. The velocity vectors in Figure 6c indicate that the medium is drawn into the scaffold due to the upward motion of the piston.

### 3.3. Mechanical Microenvironment of the Bone Layer

The post-processing results of the simulation for the bone layer of the osteochondral scaffolds with and without the barrier layer are shown in Figure 7. In certain areas of the top region of the bone layer without a barrier layer, the OSS is larger compared to other regions, as shown in Figure 7a. Nevertheless, OSS remained negligible in both bone-layer configurations, ranging from $8.18\times 10^{-10}$ to $4.76\times 10^{-5}$ in the case without a barrier layer and $9.85\times 10^{-10}$ to $2.09\times 10^{-5}$ in the case with a barrier layer. Therefore, the resulting shape deformation is insignificant (Figure 7a). In the bone layer without a barrier layer, WSS is more pronounced in the upper sublayers near the interface with the chondral layer, as shown in Figure 7b. In the barrier-layer case, the WSS is much higher in the central regions of the barrier layer, while it remains very small on the strands below it. In the bone layer, the inclusion of a barrier layer altered the WSS distribution, decreasing the minimum WSS from $1.21\times 10^{-3}\;\mathrm{mPa}$ to $4.77\times 10^{-4}\;\mathrm{mPa}$ and increasing the maximum WSS from 27.77mPa to 62.51mPa. The $WSS_{Avg}$ on the surface of the bone layer without a barrier layer is 4.31mPa, compared to 2.42mPa for the other case. This aligns with the plots of $WSS_{Avg}$ in Figure 5a, where the barrier layer reduced the average WSS on the bone layer during a loading cycle. In Figure 7c, it is evident that medium flow within the pores below the barrier layer is highly limited. Conversely, without the barrier layer, the medium can pass freely through the scaffold’s pores, particularly those located at the interface between the chondral and bone layers. All results in this figure were obtained at the moment when $S_{Max2}$ occurred (see Figure 5c), which is 0.74s for the case without a barrier layer and 0.73s for the other case.

### 3.4. Prediction of Cell Differentiation in the Chondral Layer

**Figure 8:**
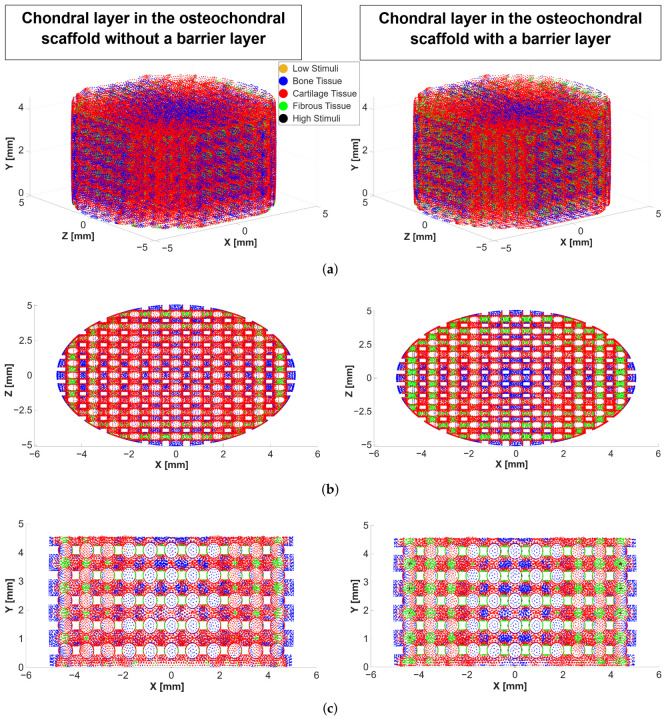
Cell differentiation on the chondral layers of the osteochondral scaffolds, both without (**left**) and with a barrier layer (**right**). The predicted cell phenotypes are visualized across the entire 3D structure of the scaffolds (**a**), from a top view (**b**), and from a side view (**c**).

Figure 8 illustrates the predicted cell differentiation due to mechanical stimulation on the surface of the chondral layer for both osteochondral scaffolds with and without a barrier layer. Each coloured point represents a cell that has differentiated from an MSC into a specific phenotype: bone, cartilage, or fibrous, indicated by the colours blue, red, and green, respectively. The top view (Figure 8b) and side view (Figure 8c) enable simpler analysis of cell distribution. Notably, the scaffold with a barrier layer exhibits a higher number of differentiated fibrous cells than the other scaffold. Additionally, the inclusion of a barrier layer enhanced bone differentiation in the central region of the scaffold (Figure 8b,c). This trend is evident from a polar-coordinate analysis: within a radius corresponding to 50% of the scaffold radius (i.e., 25% of the total scaffold surface area), 29.26% of all cells predicted to differentiate into bone cells were located in this region for the scaffold without a barrier layer. In contrast, this proportion increased to 41.13% when a barrier layer was included. In both scaffold configurations, fibrous cell differentiation was predominantly observed in the peripheral regions of the scaffold (Figure 8b,c). Specifically, within the radial region extending from 50% to 100% of the scaffold radius, 96.95% and 97.64% of all cells predicted to differentiate into fibrous tissue were located in the scaffolds without and with a barrier layer, respectively. The differentiation results depicted here are calculated at the moment when $S_{Max2}$ occurred (see Figure 5c).

### 3.5. Prediction of Cell Differentiation in the Bone Layer

**Figure 9:**
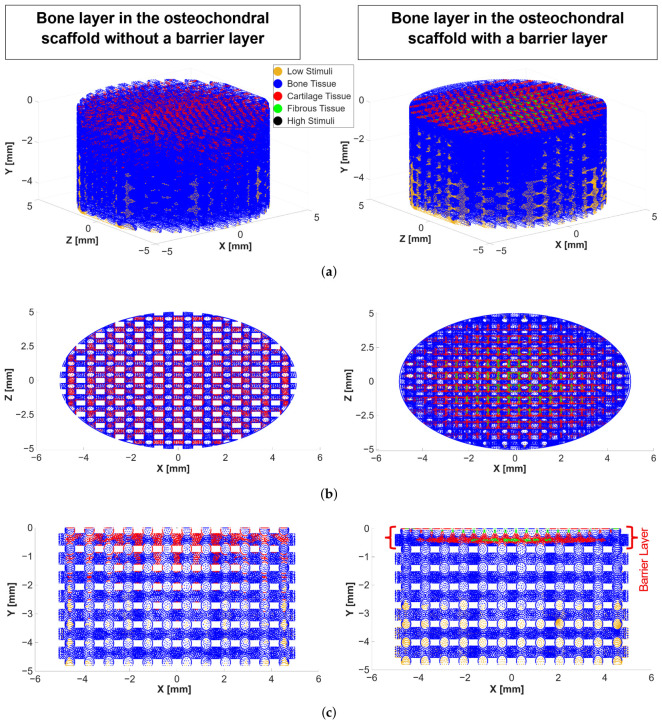
Cell differentiation on the bone layer of the osteochondral scaffolds, both without (**left**) and with a barrier layer (**right**). The predicted cell phenotypes are visualized across the entire 3D structure of the scaffolds (**a**), from a top view (**b**), and from a side view (**c**).

Cell differentiation prediction on the surface of the bone layer of the osteochondral scaffolds, both with and without a barrier layer, is shown in Figure 9. In both scenarios depicted in Figure 9a,c, it is evident that cell differentiation tends toward bone formation. Regions with very low stimuli for cell differentiation are marked in yellow. These regions are more noticeable in the scaffold with the barrier layer compared to the one without, as shown in Figure 9a,c. Cells on the upper strands’ layers of the scaffolds near the chondral interface are more stimulated, leading to their development into cartilage and even fibrous tissue (Figure 9c). To further quantify the vertical distribution of cell differentiation, the bone layer was divided into seven equally sized slices along the vertical direction (Y axis in Figure 9). Analysis revealed that the uppermost one-seventh of the bone layer contained 63.25% and 100% of all cells predicted to differentiate into cartilage cells for the scaffolds without and with a barrier layer, respectively, as qualitatively illustrated in Figure 9c. Furthermore, in the scaffold with a barrier layer, this uppermost slice also contained 100% of all cells predicted to differentiate into fibrous tissue. In contrast, no fibrous differentiation was observed in the bone layer of the scaffold without a barrier layer. The top views reveal a denser cell distribution in the bone layer with the barrier layer, with some central areas favouring fibrous tissue formation (Figure 9b). In the bone layer of the scaffold with a barrier layer, more than 74% of all cells predicted to differentiate into fibrous tissue were concentrated within the radial region extending from the inner half (0–50%) of the scaffold radius (Figure 9b). All results are calculated at the time corresponding to the occurrence of $S_{Max2}$ (see Figure 5c).

### 3.6. Quantitative Analysis of the Barrier Layer Effects on Predicted Cell Phenotypes

**Figure 10:**
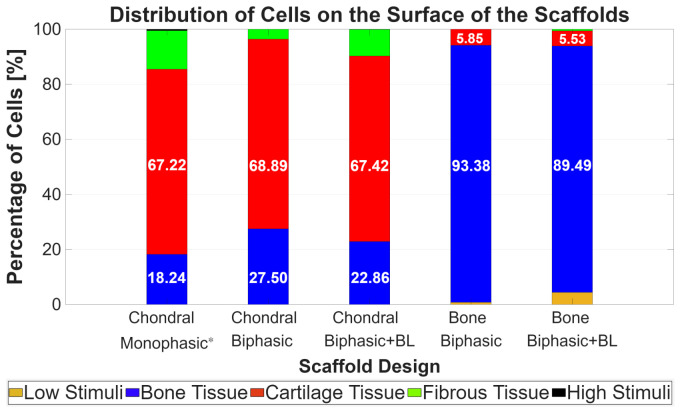
An overview of the percentage of cell differentiation on the entire surfaces of the osteochondral scaffold layers, with and without a barrier layer. On the x-axis, “Chondral Monophasic” denotes the reference monophasic cartilage scaffold. “Chondral Biphasic+BL” and “Bone Biphasic + BL” represent the chondral and bone layers of the osteochondral scaffold with a barrier layer, respectively. “Chondral Biphasic” and “Bone Biphasic” correspond to the chondral and bone layers of the osteochondral scaffold without a barrier layer. The numbers in the centre of the blue and red columns represent the percentages of cells differentiated into bone and cartilage, respectively. * Results for the monophasic cartilage reference scaffold were obtained from the previous numerical study, in which the design of a scaffold was optimised to enhance chondrogenic differentiation [25].

Finally, to provide a clear overview of cell differentiation on the osteochondral scaffolds, Figure 10 presents the percentage of MSCs predicted to differentiate into specific cell phenotypes in response to mechanical stimulation across the entire scaffold surface. The analysis includes the separate layers of the osteochondral scaffold for both cases, with and without a barrier layer. Scaffolds in the case with a barrier layer include “BL” in their name, and the monophasic cartilage reference scaffold that demonstrated the best structural design for supporting cartilage differentiation in our previous study is named “Chondral Monophasic” [25]. From this figure, several conclusions can be drawn: First, adding a barrier layer to the bone layer reduced bone cell differentiation by −3.89% due to very low stimuli in some areas. Second, the inclusion of a barrier layer slightly reduced cartilage cell differentiation in the chondral layer by −1.47%. Increasing stimuli in some areas of this layer reduced bone tissue formation by −4.64% and promoted fibrous tissue formation by +6.02%. Third, comparing the chondral layer of the biphasic scaffold without a barrier layer to the monophasic cartilage reference scaffold (Chondral Monophasic in the figure) shows a slight increase in cartilage differentiation (+1.67%) and a significant increase in bone differentiation (+9.26%), along with a reduction in fibrous tissue formation (−10.42%).

## 4. Discussion

In this work, we simulated the influence of dynamic mechanical stimulation on the early stage of cell differentiation on a cell-seeded biphasic osteochondral scaffold. Two scenarios were considered: scaffolds with and without an interfacial barrier layer. In the absence of a barrier layer, 93.38% of MSCs located on the surface of the bone layer were predicted to differentiate into bone cells, whereas 68.89% of MSCs on the chondral layer were predicted to undergo chondrogenic differentiation (Figure 10). With the incorporation of a barrier layer, these values changed to 89.49% and 67.42%, respectively (Figure 10). The simulation results showed that cell differentiation in both layers of the osteochondral scaffold progressed toward the expected lineage. Incorporating a barrier layer into the model resulted in a slight reduction in the predicted differentiation toward bone in the bone layer and toward cartilage in the chondral layer. However, this deviation was small, suggesting that the introduction of a barrier layer, commonly implemented in 3D-printed scaffolds to maintain distinct biological environments between cartilage and bone [2], has only a minor effect on cell differentiation.

### 4.1. Compressive and Fluid-Induced Shear Stimulation in the Chondral Layer

Mechanical stimulation comprised scaffold deformation and compression-induced fluid WSS. The compression force applied to the osteochondral scaffold deformed the soft material, i.e., the hydrogel-based chondral layer, while the rigid bone layer, made of PCL, remained almost undeformed. This is shown in Figure 5b, where OSS values for the soft and rigid layers are compared. The OSS on the surface of the chondral layer is primarily observed at the intersections of the strands, with values ranging from 0.03 to 0.07 (Figure 6a). A previous study employing the boundary element method (BEM) to compute surface OSS under compressive loading reported similar findings, showing that an architecture with a 90° angle between successive layers (0/90 scaffold) exhibits OSS predominantly at strut intersections, typically in the range of 0.03 to 0.1 [40]. However, that study considered a different scaffold design with a rectangular cross-section and linear elastic material properties under a compressive load of 10MPa. Elevated mechanical shear strains at strand intersections within the chondral layer promote localised cartilage and fibrous differentiation, as illustrated in Figure 8c. Similar strain concentrations under compressive loading, along with associated predictions of cartilage and fibrous tissue formation, have been reported in micro-CT-based scaffold models [41]. The WSS values in the chondral layer are higher in the presence of a barrier layer compared to the case without a barrier layer, and the corresponding WSS profile more closely resembles that of the monophasic cartilage reference scaffold optimised for cartilage regeneration, as shown in Figure 5a. This behaviour can be attributed to the barrier layer, which reduces pore size at the interface and restricts fluid flow from the chondral layer to the bone layer (Figure 6c). As a result, the flow conditions become similar to those in the monophasic cartilage reference scaffold, where the scaffold rested on the bottom of the well plate, thereby restricting flow through its underside [25]. The WSS contours in Figure 6b indicate higher values at the peripheral surfaces of both chondral layers compared to the inner regions. A similar trend was reported in a previous FSI study of a 3D micro-CT reconstructed scaffold [42]. In another study on cartilage tissue engineering, FSI modelling of regular, CAD-generated scaffolds also demonstrated that WSS varies spatially, with higher values at the periphery and lower values in the central regions [43]. As a result of increased fluid-induced stimulation in these peripheral regions, a shift toward fibrous differentiation is observed away from the central regions of the chondral layer (Figure 8). This observation is consistent with the results presented in Section 3.4, where more than 96% of all cells predicted to undergo fibrous differentiation were located within the radial region extending from 50% to 100% of the scaffold radius.

### 4.2. Compressive and Fluid-Induced Shear Stimulation in the Bone Layer

**Figure 11:**
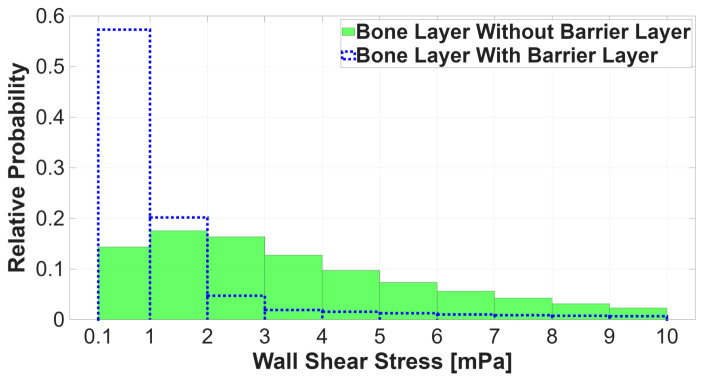
Distribution of WSS on the surfaces of the bone layers within the range of 0.1–1 mPa. Approximately 94% of MSCs on the surface of the bone layer without a barrier layer (filled green) falls within this range, whereas this proportion decreases to 90% in the presence of a barrier layer (dotted blue).

On the bone layer, the average WSS is lower in the presence of a barrier layer compared to the case without it, as shown in Figure 5a. This reduction is attributed to the barrier layer, which limits the distribution of fluid flow within the bone layer, as illustrated on the right side of Figure 7c compared to the left. In the configuration with a barrier layer, the fluid is forced to pass through smaller pores at the interface between the two scaffold layers, resulting in locally elevated WSS values at the interface (Figure 7b). The effect of increased fluid-induced stimulation at the interfacial layer is reflected by the accumulation of cartilage and even fibrous tissue formation in this region, as shown on the right side of Figure 9. This finding aligns with the results presented in Section 3.5, where the analysis of the uppermost one-seventh of the bone layer, corresponding to the location of the barrier layer, revealed that all cells predicted to differentiate into cartilage and fibrous tissue were concentrated within this region of the scaffold. For the bone layer without a barrier layer, the velocity vectors in Figure 7c (left) indicate that the fluid primarily flows through the upper regions of the layer. Consequently, higher WSS values are observed in these areas, as shown in Figure 7b (left). This results in increased fluid-induced stimulation in the top region of the bone layer, leading to cartilage cell differentiation in these areas, as depicted in Figure 9 (left). The cell differentiation results for the bone layer without a barrier layer further support this observation, as more than 90% of all cells predicted to differentiate into cartilage cells were located within the uppermost one-third of the bone layer. To examine the dominance of fluid-induced stimulation at the surface of the bone layer, the distribution of WSS is calculated and presented in Figure 11. Previous studies have reported that WSS levels in the range of 0.1–10mPa are sufficient to stimulate MSCs toward osteogenic differentiation [19,38]. For the bone layer without a barrier layer, the relative probability of MSCs experiencing WSS in this range is approximately 0.94. When a barrier layer is added, it decreases slightly to 0.9. These probabilities are slightly above the percentage of cells differentiating into bone shown in Figure 10. This difference likely results from the minor contribution of mechanical deformation to the overall stimulus, leading to partial cartilage differentiation. Furthermore, as illustrated in Figure 11, the WSS distribution within the bone layer with a barrier layer is more concentrated in narrower ranges. For example, the relative probability of MSCs experiencing WSS between 0.1–1mPa is roughly 0.57 when a barrier layer is present, compared to about 0.14 without one (Figure 11).

### 4.3. Relative Contributions of Compressive and Fluid-Induced Shear Stimulation

To assess the relative contributions of mechanical deformation and fluid-induced shear, the proportions of stimulation from OSS and WSS in determining the maximum stimulus during a loading cycle ($S_{Max2}$) were compared. For the chondral layer without a barrier layer, OSS and WSS contributed 50.81% and 49.19%, respectively, to the total stimulus, whereas upon inclusion of a barrier layer, these contributions changed to 43.46% and 56.54%, respectively. This indicates that, in the absence of a barrier layer, the chondral layer receives balanced contributions from fluid shear stress and strain-induced mechanical stimulation, whereas the inclusion of a barrier layer slightly increases the relative contribution of fluid shear stress to the overall stimulus. This analysis for the bone layer, in both cases with and without a barrier layer, revealed that WSS accounts for approximately 99.99% of the total stimulus. This finding suggests that, in the bone layer, mechanical stimulation is dominated by fluid shear stress, while strain-induced mechanical deformation has a negligible effect. Consequently, mechanical deformation primarily affects cell differentiation in the cartilage layer.

### 4.4. Dynamic Mechanical Loading Strategy and FSI Coupling Strategy

Mechanical compression was applied as a sinusoidal displacement with an amplitude of 2.5%. Owing to the high stiffness of the bone layer, the imposed displacement was accommodated almost entirely by the chondral layer, resulting in an effective compression amplitude of 5%. This corresponded to the compression applied to the monophasic cartilage reference scaffold in our previous study [25], thereby allowing the previously computed optimal mechanical stimulation condition to be transferred to the chondral region of the osteochondral scaffold. The application of pulsatile, time-dependent compression more accurately replicates physiological loading conditions and yields promising predictions of cell differentiation in the subchondral bone region (Figure 10). In a recent CFD study of different gyroid scaffolds, a transient, pulsatile, sinusoidal inlet flow was applied to compare WSS variations during fluid perfusion with those under steady flow [44]. The study revealed that time-dependent fluctuations in WSS and pressure more closely reflect physiological conditions experienced during daily activities.

While the present study employed a one-way FSI approach, its applicability is justified by the negligible influence of fluid-induced stresses on the scaffold deformation. In the investigated compressive stimulation setup, fluid flow was driven by externally imposed piston displacement, and the resulting fluid pressures were much lower than the mechanically induced stresses in the scaffold. In contrast, in systems such as perfusion bioreactors, where fluid pressure is the primary driver of scaffold deformation, the resulting structural deformation may significantly alter the flow field. Under such conditions, a two-way FSI approach should be considered.

### 4.5. Experimental Studies

Although the present study is based on a computational framework with theoretical assumptions and lacks experimental validation, the predicted region-specific differentiation patterns can be discussed in the context of previous experimental studies on osteochondral scaffold regeneration. Such comparisons do not provide direct validation of the present model because the experimental studies differ in scaffold material, geometry, biological conditions, defect models, and outcome measures. Nevertheless, they provide valuable qualitative reference points for assessing whether the predicted cartilage- and bone-forming tendencies are biologically plausible.

A relevant experimental study developed a slotted decellularised osteochondral scaffold and demonstrated enhanced cartilage and subchondral bone regeneration in a rabbit osteochondral defect model [45]. Osteochondral repair was evaluated macroscopically and histologically at 6 and 12 weeks after implantation. After 12 weeks, the scaffold functionalized with collagen-binding growth factors achieved a mean histological score corresponding to approximately 85.87% of the maximum possible score, indicating a high degree of osteochondral regeneration. This observation is qualitatively consistent with the average osteochondral differentiation predicted on the scaffold surface without a barrier layer in the present study (81.14%), defined as the mean of the chondrogenic differentiation on the chondral layer and osteogenic differentiation on the bone layer. However, the comparison remains indirect, as the experimental histological score and the numerical differentiation outcome are fundamentally different measures of osteochondral regeneration.

Another noteworthy experimental study by Sartori et al. [46] investigated a bi-layered scaffold made from collagen and magnesium-doped hydroxyapatite for osteochondral tissue regeneration using human MSCs. In vivo histological analyses demonstrated the formation of mature cartilage-like and bone-like tissues, good integration with the surrounding tissue, and preservation of the scaffold structure. After 8 weeks, the reported Boden score of approximately 14/21 for the engineered scaffold indicates a good, although not complete, degree of osteochondral repair. It should be noted, however, that the scaffold geometry, fabrication method, dimensions (⌀2mm,h=3mm), biological conditions, and outcome measurements differed substantially between the two studies.

The long-term regenerative outcomes and overall performance of osteochondral scaffolds can only be fully assessed through in vivo investigations. Therefore, the present computational model cannot directly evaluate long-term tissue regeneration or integration. Nevertheless, the region-specific differentiation patterns predicted in this study are qualitatively consistent with findings from previous in vivo studies that reported successful osteochondral regeneration, effective tissue integration, and high histological repair scores. Confidence in the biological trends predicted in this study is supported by the use of an experimentally derived and widely adopted mechanoregulation theory [37], numerical verification through mesh and time-step sensitivity analyses, and the physical consistency of the predicted mechanical stimulation patterns with the scaffold architecture and material properties.

### 4.6. Limitations and Future Research

Articular cartilage exhibits viscoelastic properties that are essential for absorbing and distributing loads, enabling deformation under mechanical loading and recovery after unloading [23]. We used a hyperelastic material model of the ADA-GEL in the chondral layer, which aligned well with experimental measurements [34]. However, applying a hyper-viscoelastic material model [47] to the chondral layer will improve the prediction of cell differentiation on the scaffold, since its material characteristics are time-dependent and change over loading cycles. Previous studies on ADA-GEL indicate that its mechanical response is time-dependent but predominantly elastic. Weizel et al. [47] identified hyper-viscoelastic parameters for ADA-GEL and showed that, although the material exhibits stress relaxation, hysteresis, and conditioning under cyclic loading, its response is dominated by elastic rather than viscous effects and relaxes faster than native articular cartilage. Complementarily, Abroug et al. [48] demonstrated that the long-term mechanical behaviour of ADA-GEL and related alginate–gelatin hydrogels is governed by coupled swelling, cross-linking, polymer–solvent interactions, bound water, and network structure; in particular, hysteresis was linked to energy dissipation and the average mesh size of the hydrogel network. Therefore, the hyperelastic material description used in the present single-cycle simulation represents a reasonable first approximation. Moreover, future work should include modelling the evolution of pore morphology due to neo-tissue formation [19], with subsequent validation through in vitro experiments. Consequently, the model was limited to the homeostatic state of the scaffold, while transient intermediate conditions that may affect cell differentiation were neglected. Incorporating such effects would require experimental data to update the scaffold morphology and material properties after a certain number of loading cycles, thereby capturing the evolving tissue–scaffold interactions over time. In addition, recent developments in computational mechanobiology have involved the use of machine learning (ML) algorithms to address complex optimisation challenges. For instance, a recent study implemented an ML-based reduced-order model within a mechanobioregulatory framework to enhance composite scaffold performance, considering both structural shear strain and WSS [49]. Another study predicted permeability in patient-specific 3D-printed bone scaffolds using a hybrid CFD–ML framework and enabled topology-aware optimisation subject to transport and mechanical constraints [50]. Future studies could also integrate ML models to optimise osteochondral scaffold architectures and their mechanical and fluidic microenvironment to further enhance osteochondral regeneration. Finally, although a barrier layer with material properties similar to those of the bone layer provides a simplified geometric representation of the osteochondral unit, future research should model an intermediate layer, primarily composed of calcified cartilage, to more accurately replicate the native layered structure of osteochondral tissue [2]. Nevertheless, a dense biomimetic calcified interface is better suited to static in vitro culture systems or in vivo studies, where its role in preserving tissue-specific biological microenvironments, preventing vascular invasion into the cartilage layer, and inhibiting cartilage calcification can be assessed more comprehensively [24].

Although the Prendergast theory [37] has been successfully applied to fracture healing, osteochondral defect healing, distraction osteogenesis, bone–implant interfaces, and tissue engineering applications, its applicability is primarily limited to mechanically regulated differentiation and the early stages of tissue formation. The model assumes that MSC fate is determined solely by local mechanical stimuli and that differentiation occurs immediately once cell fate has been determined, without explicitly accounting for the time lag between mechanosensing and differentiation or the possibility that cells migrate to a different microenvironment before differentiating [39].

Another limitation of the present study is that the conclusions are derived from a specific biphasic scaffold geometry, material combination, and loading configuration. Nevertheless, the underlying stimulation mechanisms are expected to be transferable to comparable osteochondral scaffold systems, provided that similar mechanical and structural conditions are fulfilled. In many osteochondral scaffold concepts, the bone region is designed as a relatively stiff load-bearing phase, whereas the cartilage region is composed of a softer and more deformable hydrogel-based phase. Under compressive loading, such a material contrast generally causes the applied displacement to be accommodated primarily by deformation of the chondral layer, while deformation of the bone layer remains limited. Consequently, strain-induced mechanical stimulation, particularly OSS, is expected to be substantially higher in the chondral layer than in the bone layer. This preferential deformation of the cartilage phase supports the intended stimulation toward chondrogenic differentiation, whereas the stiffer bone layer is primarily stimulated by compression-induced interstitial fluid flow and the resulting WSS, which can promote osteogenic differentiation. Since WSS was present in both scaffold regions in the current model, fluid-induced stimulation contributed to lineage-specific differentiation in both the chondral and osseous layers. Therefore, the proposed simulation approach should also be applicable to other open-porous osteochondral scaffolds for optimising loading parameters, scaffold architecture, pore morphology, and interfacial design. However, this transferability requires that the scaffold exhibit a mechanically distinct soft chondral layer and a stiff bone layer, and that its pore network permit fluid flow through both phases. For scaffold systems with markedly different material behaviour, closed or poorly interconnected porosity, strong viscoelastic effects, or substantial tissue formation during culture, the model assumptions would need to be adapted.

## 5. Conclusions

This study demonstrated the applicability of an FSI model coupled with a mechanoregulation algorithm for predicting mechanically induced early-stage cell differentiation in biphasic osteochondral scaffolds subjected to dynamic compressive loading (1 Hz, 2.5% strain). The simulations predicted region-specific differentiation patterns, with approximately 68.9% cartilage differentiation in the chondral layer and 93.4% bone differentiation in the bone layer. Compared with the scaffold without a barrier layer, the inclusion of a geometric barrier layer slightly reduced the predicted cartilage and bone differentiation by approximately 1.5% and 3.9%, respectively. Despite this small reduction, incorporating a biomimetic barrier layer remains important for 3D-printed biphasic or multiphasic osteochondral scaffolds, as its role in preserving tissue-specific biological environments has been demonstrated in previous in vitro and in vivo studies. Overall, the proposed computational framework provides a useful tool for quantitatively comparing osteochondral scaffold designs and their mechanically induced differentiation patterns. However, the results should be interpreted as computational predictions until the model has been experimentally validated. The framework can then be extended by integrating time-dependent material behaviour, tissue growth, and remodelling, thereby improving its predictive capability and extending its applicability to long-term osteochondral regeneration.

## Figures and Tables

**Table 1:** Classifying tissue phenotype is defined by the computed value of the stimuli (S).

S≤0.01	0.01<S≤1	1<S≤3	3<S≤6	S>6
Very low stimuli	Bone tissue differentiation	Cartilage tissue differentiation	Fibrous tissue differentiation	Very high stimuli

**Figure A2:**
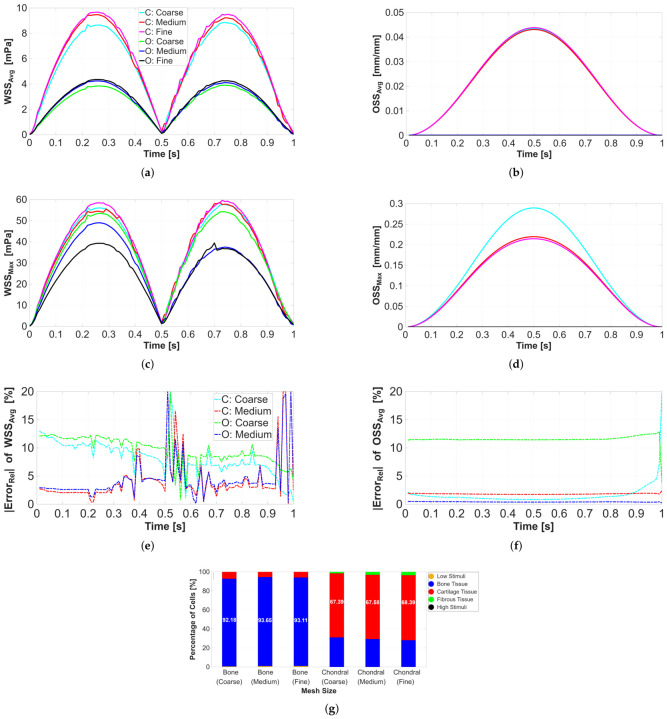
The influence of mesh size on: (**a**) $WSS_{Avg}$ on the surface of the chondral layer (shown in the legend as C) and the bone layer (indicated in the legend by O), (**b**) $OSS_{Avg}$ on the surface of the chondral and bone layer, (**c**) $WSS_{Max}$ on the surface of the chondral and bone layer, (**d**) $OSS_{Max}$ on the surface of the chondral and bone layer. (**e**) Magnitude of the relative error of $WSS_{Avg}$ for coarse and medium meshes compared to the fine mesh in both the chondral and bone layers. (**f**) Magnitude of the relative error of $OSS_{Avg}$ for coarse and medium meshes relative to the fine mesh in both the chondral and bone layers. (**g**) Cell phenotype prediction under mechanical stimulation for the bone and chondral layers using coarse, medium, and fine meshes. The percentages within the blue and red bars indicate the predicted bone formation in the bone layer and cartilage formation in the chondral layer, respectively.

## Data Availability

The simulation data supporting the conclusions of this article will be made available by the corresponding author on request.

## Abbreviations

The following abbreviations are used in this manuscript:

3D	Three-dimensional
ADA-GEL	Alginate–gelatin
BEM	Boundary element method
BL	Barrier layer
CAD	Computer-aided design
CFD	Computational fluid dynamics
CT	Computed tomography
DIW	Direct ink writing
$D_{Scaffold}$	Scaffold diameter
$D_{Strand}$	Strand diameter
FE	Finite element
FSI	Fluid–structure interaction
h	Vertical span
$H_{Scaffold}$	Scaffold height
ML	Machine learning
MSC	Mesenchymal stem cell
OSS	Octahedral shear strain
PCL	Polycaprolactone
S	Stimuli
WSS	Wall shear stress
Y	Horizontal span

## Appendix A

### Appendix A.2. Mesh Generation

To obtain a sufficient number of elements, a mesh-independence study was conducted for the osteochondral scaffold without a barrier layer. Three mesh configurations, i.e., coarse, medium, and fine meshes, were considered. The influence of element size on $WSS_{Avg}$, $OSS_{Avg}$, $WSS_{Max}$, and $OSS_{Max}$ for both chondral and bone layers is shown in Figure A2a–d. Relative error in the $WSS_{Avg}$ and $OSS_{Avg}$ obtained with the coarse and medium meshes compared with the fine mesh for the chondral and bone layers are shown in Figure A2e,f. The maximum relative error shown in Figure A2e,f is limited to 20% to improve the visibility of the curves. The time points at which the relative error exceeded this limit correspond to very small values of $WSS_{Avg}$ or $OSS_{Avg}$, leading to an increase in the relative error despite negligible absolute differences between the meshes. For example, the large relative error in $WSS_{Avg}$ near t=0.5s occurs because the piston reaches its lowest position, resulting in wall shear stress values close to zero over the scaffold surface. The relative error of the medium mesh remained below 5% for $WSS_{Avg}$ throughout most of the simulation in both the chondral and bone layers (Figure A2e). For $OSS_{Avg}$, the relative error was below 2% for most of the simulation time (Figure A2f). Furthermore, cell phenotype predictions on the scaffold surfaces (Figure A2g) show that the medium mesh has an absolute error of less than 1% for both bone and chondral layers compared to the fine mesh. Consequently, the medium mesh configuration was chosen for the simulations of both osteochondral scaffolds, with and without a barrier layer.

The selected mesh includes 4,316,652 elements in the solid domain and 4,603,685 in the fluid domain. Mesh details for both FE and CFD models are illustrated in Figure 3. The element size on the scaffold surface was set at 0.2 mm with a curvature normal angle of 27°. In other regions of the solid and fluid domains, the element sizes and curvature normal angles were 0.6 mm and 67°, respectively. The scaffold and piston in the FE model used SOLID187 elements, whereas the support used SOLID186 elements. Using SOLID187 elements for the scaffold was essential because they are 10-node, high-order 3D tetrahedral elements capable of managing large deformations for hyperelastic materials. Both solid and fluid domains were meshed simultaneously in ANSYS Meshing to create a conformal mesh. The model with a barrier layer has the same configuration as the one without, containing 4,654,908 elements in the solid domain and 4,514,287 in the fluid domain.

Finally, to perform the mesh sensitivity study, convergence of the finite element model at each time step was achieved by satisfying the default ANSYS Transient Structural force and displacement convergence criteria. For the CFD model, the absolute scaled residual criterion for both continuity and momentum equations was set to $1\times 10^{-4}$, with a maximum of 50 iterations per time step to ensure convergence of the flow solution. The FSI simulation employed a time step size of 0.01 s, resulting in 100 time steps over one loading cycle. In the one-way ANSYS System Coupling analysis, one coupling iteration was performed per time step. All simulations in this study utilized the setup described above.
